# Identification of DNA-Binding Factor Enrichment in Chromatin Accessibility Data to Define a Persister Cell Signature

**DOI:** 10.21769/BioProtoc.5800

**Published:** 2026-08-20

**Authors:** Vidya Ajay, Mihai G. Dumbrava, Alexandre Gaspar-Maia, Wazim Mohammed Ismail

**Affiliations:** 1 Division of Experimental Pathology, Department of Lab Medicine and Pathology, Mayo Clinic, Rochester, MN, USA; 2 Mayo Medical Scientist Training Program, Mayo Clinic, Rochester, MN, USA

**Keywords:** Chromatin accessibility, Multiome, snATAC-seq, ChIP-seq, CUT&RUN, Single cell, Epigenomics

## Abstract

Chemotherapy-resistant persister cells are a major driver of cancer recurrence, yet their epigenetic basis remains poorly characterized. This protocol describes a computational pipeline for identifying DNA-binding factors (DBFs) that are enriched in accessible chromatin that collectively define a persister cell signature (PCS). Starting from single-nucleus ATAC-seq (snATAC-seq) data processed through the 10x Genomics CellRanger ARC pipeline, this protocol covers (1) the creation of a Seurat/Signac object with ATAC peaks, (2) the optional integration of DNA-binding data from the ReMap2022 database as a per-cell chromatin module assay, (3) differential accessibility analysis across clinically defined comparison groups, and (4) identifying and defining the top enriched DBFs as the PCS. This approach is applicable to any snATAC-seq dataset in which cells can be grouped by clinical response, treatment status, or resistance phenotype.

Key features

• Integrated analysis of chromatin accessibility data with publicly available DNA-binding data is a useful technique to identify potential epigenetic biomarkers.

• Single-nucleus ATAC-seq from clinically relevant samples can be used to identify binding enrichment of specific DNA-binding factors in sub-populations of cells.

• This protocol was used to identify a persister cell signature that was able to predict chemotherapy resistance in high-grade serous ovarian cancer.

## Background

Understanding the molecular mechanisms that drive cancer initiation, progression, and therapeutic resistance is crucial for devising effective therapies for cancer treatment. In addition to the genetic drivers of cancer, such as somatic mutations, copy number variations, and genomic instability, new research has shown epigenetic mechanisms as potential drivers of cancer progression and therapeutic resistance [1–3]. These epigenetic features modulate transcriptional programs and cellular functions through a variety of mechanisms without modifying the underlying DNA sequence. Underlying these epigenetic mechanisms is ultimately the binding of transcription factors (TF) and chromatin remodelers at specific non-coding regions of the DNA known as regulatory elements. These elements, such as promoters, enhancers, or silencers, regulate gene expression when engaged by specific TFs [4]. Using chromatin accessibility [5] to infer TF binding at these regulatory regions allows for a more comprehensive understanding of expression regulation.

The single-nucleus assay for transposase-accessible chromatin by sequencing (snATAC-seq) [5,6] performs high-throughput profiling of chromatin accessibility at single-cell resolution, allowing us to identify cell-specific epigenetic patterns. By combining this information with publicly available ChIP-seq and CUT&RUN data for currently available DNA-binding factors (DBFs) (TFs and chromatin regulators) [7], we can predict the enrichment of potential binding events of each factor in any given cell using in silico methods such as ChromVAR [8] and ReMapEnrich. This approach allows us to characterize unique epigenetic signatures that link chromatin accessibility to DBFs, enabling the discovery of key transcription factors or chromatin regulators as targetable epigenetic biomarkers [9,10].

In our study aimed at characterizing the chromatin landscape and transcriptional features of drug-tolerant persister cells in high-grade serous ovarian cancer (HGSOC), we performed single-nucleus multiomic profiling (snRNA+snATAC seq) of treatment-naïve and neoadjuvant chemotherapy (NACT)-treated tissues from patients with HGSOC [11]. By comparing the open-chromatin signatures between the treatment-naïve patients who later developed resistance to adjuvant chemotherapy and the NACT-treated patients, we identified a distinct epigenetic signature of persister cells that distinguished the chemotherapy response in treatment-naïve tumors.

In this paper, we describe the computational protocol that was developed to identify this persister cell signature using chromatin accessibility data from snATAC-seq. The protocol starts with snATAC-seq data and a database of DNA-binding information (ChIP-seq/CUT&RUN data) formatted as a four-column BED file. We used data downloaded from ReMap 2022 for this purpose. We assume that the snATAC-seq count matrices from all the samples in the cohort are already merged/pooled and that the cell types are previously identified and included as a metadata table. In the current example of HGSOC, we have three specific groupings of samples. First, the samples are grouped as naïve and NACT. The naïve group is further subdivided into two groups: sensitive and resistant. The snATAC-seq data is initially processed using Signac [12–14] with TF-IDF normalization and singular value decomposition for dimensionality reduction. We then incorporate two different methods to combine DNA-binding information with snATAC-seq data. The first method uses ChromVAR to calculate the enrichment score (bias-corrected deviation score) for each DBF and for each cell. These scores can be used to calculate differential enrichment between any two groups of cells. In the second method, we use ReMapEnrich to calculate the ranked enrichment of DBFs in a single set of open-chromatin regions. This method is specifically used on the open-chromatin regions that were simultaneously up-regulated in the naïve-resistant cohort and in the NACT cohort (both comparisons using the naïve-sensitive cohort as control), to define the persister cell signature. We believe that the protocol will serve as guidance for researchers interested in multimodal analysis of snATAC-seq data in combination with a database of DNA-binding data, such as ChIP-seq or CUT&RUN. In addition, we also describe using gene expression data from snRNA-seq to filter DBFs that are lowly expressed in the cell population of interest.

## Software and datasets

**Table:** 

Type	Software/dataset/resource	Version	Date	License	Access (free or paid)
Data	ReMap2022 non-redundant peaks BED file	4	2022	CC BY-NC 4.0	Free; https://remap.univ-amu.fr/download_page
Software	R	v4.5.2	2025	GPL	Free; https://www.r-project.org/
Software	Seurat	v5.4.0	2025	MIT	Free; https://satijalab.org/seurat/
Software	Signac	v1.16.0	2025	MIT	Free; https://stuartlab.org/signac/
Software	BPCells	v0.3.1	2025	MIT	Free; https://github.com/bnprks/BPCells
Software	chromVAR	v1.32.0	2025	MIT	Free: https://github.com/GreenleafLab/chromVAR
Software	ReMapEnrich	v0.99.0	2026	MIT	Free: https://github.com/remap-cisreg/ReMapEnrich
Software	ggplot2	v4.0.3	2026	MIT	Free
Software	bedtools	v2.31.0	2023	GPL	Free https://bedtools.readthedocs.io/en/latest/

## Procedure

*Note: All the required code is available at* *https://github.com/LabFunEpi/pcs_protocol* *under the MIT license.*

**A. Read counts data and prepare the Seurat object**

1. Start an R or RStudio session and set the working directory to the folder where the input data and files are located.

setwd("~/Desktop/pcs_protocol")

2. To run this protocol, we require some software packages to be installed and loaded.

*Note: If any of the packages below are not installed, usually one of the following commands can be used to install them. For more information on how to download a specific package, please check its documentation.*

The commands to install all required packages are as follows:

# Prerequisites

install.packages("BiocManager")

install.packages("remotes")

# Specify Bioconductor release 3.22 and install Bioconductor packages

BiocManager::install(version = "3.22")

BiocManager::install(c(

 "BSgenome",

 "GenomicRanges",

 "GenomeInfoDb",

 "BSgenome.Hsapiens.UCSC.hg38",

 "chromVAR",

 "motifmatchr"

))

# CRAN packages

remotes::install_version("readr", version = "2.2.0")

remotes::install_version("Seurat", version = "5.4.0")

remotes::install_version("Signac", version = "1.16.0")

remotes::install_version("data.table",version = "1.18.2.1")

remotes::install_version("dplyr", version = "1.2.1")

remotes::install_version("tidyr", version = "1.3.2")

remotes::install_version("Matrix", version = "1.7-5")

remotes::install_version("magrittr", version = "2.0.5")

remotes::install_version("stringr", version = "1.6.0")

remotes::install_version("tidyverse", version = "2.0.0")

# GitHub packages

remotes::install_github("bnprks/BPCells/r", ref = "v0.3.1")

remotes::install_github("remap-cisreg/ReMapEnrich", ref = "cb46422")

Once the packages are installed, load them into the session by running the following commands:

library(BPCells)

library(readr)

library(BSgenome)

library(GenomicRanges)

library(GenomeInfoDb)

library(BSgenome.Hsapiens.UCSC.hg38)

library(Seurat)

library(Signac)

library(chromVAR)

library(ReMapEnrich)

library(motifmatchr)

library(data.table)

library(dplyr)

library(tidyr)

library(Matrix)

library(magrittr)

library(stringr)

library(tidyverse)

3. Input data: Most CellRanger pipelines will output an obj_feature_bc_matrix.h5 file, which is a counts matrix. Any similar .h5 file can be read directly using this command:

*Note: Replace the count_10x.h5 file name with your .h5 filename; if you are not in the same directory as the file, specify the path to the file in the filename. We use the feature_type = “Peaks” argument to extract the ATAC counts from a single-cell multiome experiment that has both RNA and ATAC counts, but if you have only an ATAC assay, you may omit it.*

mat = open_matrix_10x_hdf5("data/counts_10x.h5", feature_type = "Peaks")

4. Once the counts are loaded, read the metadata file that contains cell types and the comparison groups associated with each cell. A sample table of this comma-separated file (.csv format) with only the first two rows is shown in Table 1.

**Table 1.** Sample table showing the first two rows of metadata.csv.

celltype	grouping1	grouping2
T_cell:CD4+	Naïve	Sensitive
Epithelial_cells	NACT	Resistant

The following commands will read the metadata file and create a Seurat object with the ATAC peak counts as an assay:

md = read_csv("data/metadata.csv")

chrom_assay = CreateChromatinAssay(counts = mat, sep = c("-", "-"))

obj = CreateSeuratObject(counts = chrom_assay, assay = "ATAC", genome = "hg38")

*Note: The order of the rows in the metadata file should match the order of the cells/barcodes in the counts object.*

5. Once the Seurat object is created, add the cell type annotation and group classification from the metadata for comparison. Here, we have two groups, one for naïve vs. NACT (neo-adjuvant chemotherapy) (grouping1) and one further classification for sensitive vs. resistant within the NACT group (grouping2).

obj$celltype = md$celltype

obj$grouping1 = md$grouping1

obj$grouping2 = md$grouping2

6. Normalize the data, find highly variable features, and write the Seurat object to a file.

obj = obj %>% RunTFIDF() %>% FindTopFeatures(min.cutoff = 20) %>% RunSVD()

*Note: min.cutoff=20 in FindTopFeatures() is a threshold to filter out peaks with a total count below 20 across all cells, removing low-signal peaks from the SVD step.*

saveRDS(obj, "data/seurat_obj.rds")

**B. Prepare ReMap data**

1. Download the ReMap2022 non-redundant peaks data from https://remap.univ-amu.fr/download_page. The file should correspond to the genome that you are working with. Here, we are using hg38; the file is downloaded as remap2022_nr_macs2_hg38_v1_0.bed.gz. All of the commands in this section should be executed in a terminal window (outside R). First, navigate to the folder in which your downloaded ReMap files are using this command (replace with your filepath):

cd ~/protocol_paper/data

2. Extract the compressed BED file:

gunzip remap2022_nr_macs2_hg38_v1_0.bed.gz

3. The ReMap BED file has four tab-separated columns (chromosome, start, end, source:DBF). Clean up the fourth column, which usually has cell line or tissue information (source), to retain just the DBF name; then, remove unwanted binding sites, retaining only those in the standard chromosomes, by running these commands in the terminal (outside R).

cut -d":" -f1 remap2022_nr_macs2_hg38_v1_0.bed > remap2022_nr_macs2_hg38_v1_0_MOD.bed

grep -E '^chr([0-9]+|X|Y|M)[[:space:]]' remap2022_nr_macs2_hg38_v1_0_MOD.bed > remap2022_nr_macs2_hg38_v1_0_MOD_clean.bed

4. (Optional) You can add additional annotated peaks to the ReMap2022 BED file if you have ChIP-seq or CUT&RUN data (peaks BED file) for DBFs of interest that are not represented in the database.

a. The additional BED files should be tab-separated with these columns: chromosome, start, end, and DBF name. Here is an example BED file (addons.bed) showing only the first two rows:

chr1 9829 10459 TF1

chr1 245890 246634 TF2

b. Merge this addons.bed with the ReMap2022 BED file, then sort and clean the resulting file using this command in a terminal window (outside R):

cat remap2022_nr_macs2_hg38_v1_0_MOD.bed addons.bed | sort -k1,1 -k2,2n | grep -E '^chr([0-9]+|X|Y|M)[[:space:]]' > myremap.bed

5. (Run only if step B4 was skipped): If not using a customized ReMap BED file, run this command to rename the ReMap BED file from step B3 for consistency.

mv remap2022_nr_macs2_hg38_v1_0_MOD_clean.bed myremap.bed

**C. (Optional) Add ReMap data as an assay of DNA-binding scores in each peak**

*Note: This optional step uses the AddChromatinModule function from the Signac R package, which internally uses ChromVAR to calculate a bias-corrected deviation score (enrichment score) for each DBF per cell. These scores can be used for differential binding-enrichment analysis.*

1. Set up the genome (hg38) information and filter to keep only the standard chromosome sequences.

genome <- BSgenome.Hsapiens.UCSC.hg38

keepBSgenomeSequences <- function(genome, seqnames) {

 stopifnot(all(seqnames %in% seqnames(seqinfo(genome))))

 genome@user_seqnames <- setNames(seqnames, seqnames)

 genome@seqinfo <- genome@seqinfo[seqnames]

 genome

}

sequences_to_keep <- paste0("chr", c(1:22, "X", "Y"))

genome <- keepBSgenomeSequences(genome, sequences_to_keep)

2. Export the peaks in the ATAC assay as a BED file. The peak coordinates are stored as row names of the ATAC assay in the format chr-start-end. We parse those row names into separate columns and write them out as a tab-delimited (BED) file.

# load seurat object

obj <- readRDS("data/seurat_obj.rds")

# export peaks BED from atac assay to file

data.frame(Peak = rownames(obj)) %>%

 separate(col = "Peak", sep = "-", into = c("chr", "start", "end")) %>%

 write.table("multiome_peaks.bed", sep="\t", quote=FALSE,

 row.names=FALSE, col.names=FALSE)

3. Intersect the multiome peaks BED file against the ReMap2022 BED file using bedtools [15] intersect (run outside R in a terminal window). We use the custom BED file created in section B here.

bedtools intersect -a multiome_peaks.bed -b myremap.bed -wa -wb > peaks_x_remap.txt

*Note: peaks_x_remap.txt has seven columns, since bedtools intersect -wa -wb writes out every column from file A (the peaks from the multiome data), followed by every column from file B (ReMap peaks). A sample peaks_x_remap.txt looks like this:*

chr1 9782 10725 chr1 9829 10459 SMARCA4

chr1 9782 10725 chr1 9880 10389 RELB

chr1 9782 10725 chr1 9883 10270 MLLT1

4. Read the output file from the previous step and use it to calculate the binding enrichment scores of each DBF in the ReMap BED file for each cell. Then, add these enrichment scores as an assay (named as “remap”) in the Seurat object.

# Read the overlaps calculated by bedtools intersect

hits <- fread("peaks_x_remap.txt", col.names = c("peak", "TF"))

# Cols 1-3 are your peak coords, col 7 is the TF name from remap

hits <- hits[, .(peak = paste(V1, V2, V3, sep = "-"), TF = V7)]

hits <- hits[grepl("^chr([0-9]+|X|Y|M)$", sub("-.*", "", hits$peak))]

# Make a list of overlapping multiome peaks for each DBF

TF_features <- split(hits$peak, paste0("remap_", hits$TF))

TF_features <- TF_features[lengths(TF_features) > 0]

# Calculate the enrichments

obj <- AddChromatinModule(obj, features = TF_features,

 genome = genome, assay = "ATAC")

# Move the enrichments from the meta.data to an assay

remapscores <- obj@meta.data %>% dplyr::select(starts_with("remap-")) %>% t()

rownames(remapscores) <- str_sub(rownames(remapscores), 7L, -1L)

*Note: After t(), the row names of remapscores are prefixed with "remap-", which is seven characters, so the DBF name starts after the seventh character. We use the parameters 7L, -1L to extract the names of the DBFs from the seventh character until the last one.*

obj[["remap"]] <- CreateAssayObject(data = remapscores)

obj@meta.data <- obj@meta.data %>% dplyr::select(!starts_with("remap-"))

saveRDS(obj, "data/seurat_obj.rds")

**D. Defining the persister cell signature (PCS) peaks**

1. Load the Seurat object and set the default assay to ATAC and subset to the cell type of interest.

obj <- readRDS("data/seurat_obj.rds")

DefaultAssay(obj) <- "ATAC"

Tumor_Epi <- subset(obj, subset = celltype == "Epithelial_cells")

*Note: Subsetting to a single cell type avoids confounding variation from other cell types. Adjust the celltype to match the label in your metadata.*

2. Perform differential accessibility testing to find the differential accessible peaks in two comparisons. Here, we have the first comparison as treatment-naïve vs. neo-adjuvant chemotherapy, and the second within the treatment-naïve group as sensitive vs. resistant.

*Note: min.pct is designed to speed up FindMarkers() by skipping peaks accessible in very few cells before testing. Since speed was not a constraint here, we used min.pct=0 to ensure every peak was tested. Regardless of this choice, with the total number of tests spanning 5–6 orders of magnitude, the Bonferroni correction will effectively exclude low confidence and sparsely accessible peaks.*

daps_NaiveNACT <- FindMarkers(Tumor_Epi, ident.1 = "NACT", ident.2 = "Naive",min.pct = 0 , group.by = "grouping1") %>% rownames_to_column("peak")

write.table(daps_NaiveNACT, "results/daps_NACT_vs_Naive.tsv", sep = "\t", row.names = FALSE, col.names = TRUE, quote = FALSE)

daps_SensRes <- FindMarkers(Tumor_Epi, ident.1 = "Resistant", ident.2 = "Sensitive",min.pct = 0 , group.by = "grouping2") %>% rownames_to_column("peak")

write.table(daps_SensRes, "results/daps_Resistant_vs_Sensitive.tsv", sep = "\t", row.names = FALSE, col.names = TRUE, quote = FALSE)

*Note: Your comparison group names may differ. Make sure that the correct classifications are present in ident.1 and ident.2 in the FindMarkers() function. Seurat computes differential accessibility as ident.1 vs. ident.2, with positive values indicating upregulation in ident.1.*

3. Filter each result file to significantly upregulated peaks (adjusted p < 0.05, positive log2FC) and find the intersection of differentially accessible peaks in both comparisons. Conceptually, this extracts the peaks that are both uniquely accessible in neo-adjuvant chemotherapy cells (as compared to treatment-naïve) and further uniquely accessible in chemotherapy-resistant (as compared to chemotherapy-sensitive) cells.

NACTNaiv_P <- read.table("results/daps_NACT_vs_Naive.tsv", sep="\t", header = TRUE) %>%

drop_na() %>%

dplyr::filter(p_val_adj < 0.05 & avg_log2FC > 0) %>%

pull(peak)

ResiSens_P <- read.table("results/daps_Resistant_vs_Sensitive.tsv", sep = "\t", header = TRUE) %>%

drop_na() %>%

dplyr::filter(p_val_adj < 0.05 & avg_log2FC > 0) %>%

pull(peak)

patient_PCS_peaks <- base::intersect(NACTNaiv_P, ResiSens_P)

write.table(data.frame(peak = patient_PCS_peaks) %>%

separate(peak, c("chr", "start", "end"), sep = "-") %>%

arrange(chr, as.numeric(start)), "results/patient_PCS_peaks.bed", sep = "\t", row.names = FALSE, col.names = FALSE, quote = FALSE)

**E. Finding enriched DBFs in the PCS peaks**

1. First, define all the valid genes in the dataset based on valid gene symbols, and then run ReMapEnrich on the PCS peaks to calculate enrichment scores for DBFs in the PCS peaks and select the top 120 DBFs based on adjusted p-value as the PCS signature.

**Figure 1.**
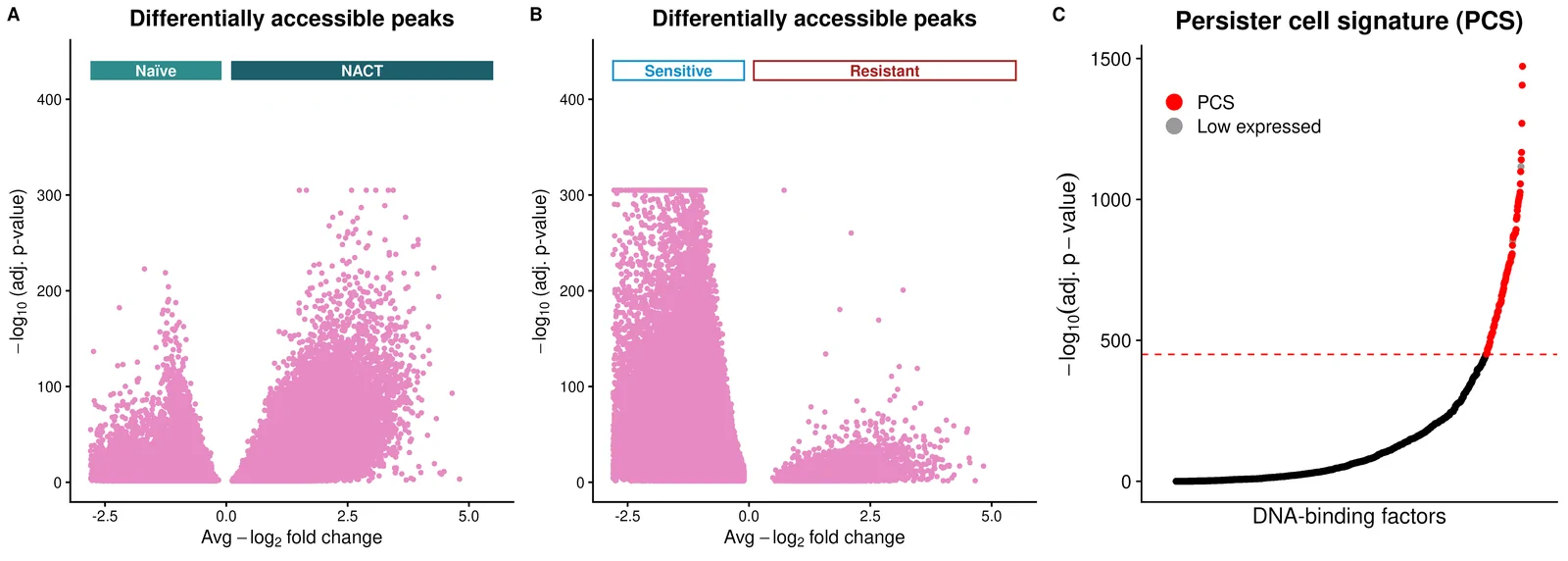
Persister cell signature (PCS) visualization. (A) Volcano plot of differentially accessible peaks in epithelial cells in the neoadjuvant chemotherapy (NACT) vs. naïve comparison. (B) Volcano plot of differentially accessible peaks in epithelial cells in the resistant vs. sensitive comparison. (C) Top 100 DNA-binding factors (DBFs) enriched in open chromatin regions common to both NACT and resistant cells (filtered for expression) are defined as the persister cell signature (PCS). Enrichment statistics were calculated using ReMapEnrich.

*Note: The threshold of 120 factors was determined empirically from the rank-order plot of enrichment significance shown in Figure 1C, selecting the inflection point beyond which lower-order factors no longer improved the accuracy of the PCS score (calculated using ModuleScore) in distinguishing the persister cell state. When applying this protocol to other datasets, users should evaluate the threshold for their own biological context, rather than assuming that n = 120 will be optimal.*

all_genes <- keys(org.Hs.eg.db, keytype = "SYMBOL")

remapCatalog <- bedToGranges("data/myremap.bed")

set.seed(1)

en <- enrichment(patient_PCS_peaks %>% StringToGRanges(), remapCatalog, chromSizes = loadChromSizes("hg38"), byChrom = FALSE)

temp <- data.frame(en) %>%

drop_na() %>%

arrange(-`q.significance`) %>%

filter(category %in% all_genes)

plotdata <- temp %>%

mutate(category = factor(category, levels = temp$category)) %>%

mutate(rn = row_number())

patient_PCS_factors <- plotdata %>% pull(category) %>% as.character()

patient_PCS_factors_120 <- plotdata %>%

pull(category) %>%

head(n = 120) %>%

as.character()

write.table(patient_PCS_factors_120, "results/PCS_ReMapEnrich.csv", row.names = FALSE, col.names = FALSE)

**F. (Optional) Filter the top 120 DBFs based on gene expression**

1. If corresponding scRNA-seq gene expression data are available, such as from a single-cell multiome experiment, we can further refine the top 120 DBFs based on gene expression. This will remove relatively low-expressed factors from the PCS list, making it more biologically relevant. The first step in this optional part is to load the scRNA counts matrix into a Seurat object.

mat = open_matrix_10x_hdf5("data/counts_10x_RNA.h5")

md = read_csv("data/metadata.csv")

obj <- CreateSeuratObject(counts = mat, assay = "RNA")

2. Add the celltype annotations to the Seurat object as metadata, just as previously done for the scATAC matrix.

obj$celltype = md$celltype

obj$grouping1 = md$grouping1

obj$grouping2 = md$grouping2

3. Subset the object to just the celltype of interest, which in this case is epithelial cells.

Tumor_Epi <- subset(obj, subset = celltype == "Epithelial_cells")

4. Calculate the percentage of NACT cells that express each gene in the subsetted object of epithelial cells.

NACT_perc_expr <- enframe(rowSums(Tumor_Epi$RNA$counts[,which(Tumor_Epi$grouping1 == "NACT")] > 0) * 100 / sum(Tumor_Epi$grouping1 == "NACT"))

5. Now we can extract the top 100 expressed PCS factors from the top 120 chromatin-enriched PCS factors to get a refined PCS list.

patient_PCS_factors_rna <- NACT_perc_expr %>% filter(name %in% patient_PCS_factors_120) %>% arrange(-value) %>% head(n = 100) %>% pull(name)

*Note: The choice of picking the top 100 expressed factors was based on the observation that DBFs expressed in <5% of the cells in the NACT group did not contribute to changes in the PCS scores and subsequent findings.*

**G. Visualization**

1. Generate a plot of all DBFs ranked by enrichment significance, highlighting the top 100 factors (PCS) in red.

library(ggplot2)

library(cowplot)

#plot 1

plotdata <- temp %>%

 mutate(category = factor(category, levels = temp$category)) %>%

 mutate(issig = case_when(

 category %in% patient_PCS_factors_rna ~ "PCS",

 category %in% patient_PCS_factors_120 ~ "Low expressed",

 TRUE ~ "Other"

 )) %>%

 mutate(issig = factor(issig, levels = c("Other", "Low expressed", "PCS"))) # PCS last = drawn on top

p1 <- ggplot(data = plotdata %>% arrange(issig), # Other first, PCS last (on top)

 mapping = aes(x = category, y = `q.significance`, color = issig)) +

 ggrastr::rasterise(geom_point(size = 2, stroke = 0.1, shape = 16), dpi = 400) +

 scale_color_manual(

 values = c("Other" = "black", "Low expressed" = "grey60", "PCS" = "red"),

 breaks = c("PCS", "Low expressed"), # only these two appear in legend

 name = NULL

 ) +

 scale_x_discrete(expand = c(0.1, 0.1)) +

 geom_hline(yintercept = 450, linetype = "dashed", color = "red", linewidth = 0.5) +

 labs(

 x = "DNA-binding factors",

 y = expression(-log[10](adj.~p-value)),

 title = "Persister cell signature (PCS)"

 ) +

 guides(color = guide_legend(override.aes = list(size = 5))) +

 theme_cowplot() +

 theme(

 axis.text.x = element_blank(),

 axis.ticks.x = element_blank(),

 legend.position = c(0.05, 0.85),

 plot.title = element_text(hjust = 0.5),

 plot.background = element_rect(fill = "white", color = NA),

 panel.background = element_rect(fill = "white", color = NA)

 )

2. Generate a volcano plot for differentially accessible peaks in the NACT vs. naïve comparison.

tbl <- read.table(file = "daps_NACT_vs_Naive.tsv", sep = "\t", skip = 1) %>%

 set_colnames(c("peak", "p_val", "effect_size", "pct.1", "pct.2", "p_val_adj"))

temp1 <- tbl %>%

 filter(p_val_adj < 0.05) %>%

 mutate(p_val_adj = ifelse(p_val_adj == 0, 1e-305, p_val_adj)) %>%

 mutate(neg_log10_adj_pval = -log10(p_val_adj))

p2 <- ggplot(temp1, aes(x = effect_size, y = neg_log10_adj_pval)) +

 ggrastr::rasterise(geom_point(data = temp1, color = "#e78ac3", size = 1.5, stroke=0.1, shape = 16), dpi = 400) +

 annotate("rect", xmin = -2.8, xmax = -0.1, ymin = 420, ymax = 440,

 fill = "#2E8B8B", color = NA) +

 annotate("text", x = -1.45, y = 430, label = "Naïve",

 color = "white", size = 3.5, fontface = "bold") +

 annotate("rect", xmin = 0.1, xmax = 5.5, ymin = 420, ymax = 440,

 fill = "#1C5F6B", color = NA) +

 annotate("text", x = 2.8, y = 430, label = "NACT",

 color = "white", size = 3.5, fontface = "bold") +

 scale_x_continuous(

 limits = c(-2.8, 5.5),

 breaks = c(-2.5, 0, 2.5, 5.0)

 ) +

 theme(axis.title = element_blank()) +

 labs(

 title = "Differentially accessible peaks",

 x = expression("Avg" ~-log[2] ~ "(adj. p-value)"),

 y = expression(-log[10] ~ "(adj. p-value)")

 )+

 theme_classic(base_size = 12) +

 theme(

 plot.title = element_text(hjust = 0.5, face = "bold"),

 axis.line = element_line(color = "black"),

 panel.grid = element_blank()

 )

3. Generate a similar volcano plot for differentially accessible peaks in the resistant vs. sensitive comparison.

tbl <- read.table(file = "daps_Resistant_vs_Sensitive.tsv", sep = "\t", skip = 1) %>%

 set_colnames(c("peak", "p_val", "effect_size", "pct.1", "pct.2", "p_val_adj"))

temp1 <- tbl %>%

 filter(p_val_adj < 0.05) %>%

 mutate(p_val_adj = ifelse(p_val_adj == 0, 1e-305, p_val_adj)) %>%

 mutate(neg_log10_adj_pval = -log10(p_val_adj))

p3 <- ggplot(temp1, aes(x = effect_size, y = neg_log10_adj_pval)) +

 ggrastr::rasterise(geom_point(data = temp1, color = "#e78ac3", size = 1.5, stroke=0.1, shape = 16), dpi = 400) +

 annotate("rect", xmin = -2.8, xmax = -0.1, ymin = 420, ymax = 440,

 fill = NA, color ="#128ecc" ) +

 annotate("text", x = -1.45, y = 430, label = "Sensitive",

 color = "#128ecc", size = 3.5, fontface = "bold") +

 annotate("rect", xmin = 0.1, xmax = 5.5, ymin = 420, ymax = 440,

 fill = NA, color = "#a62323") +

 annotate("text", x = 2.8, y = 430, label = "Resistant",

 color = "#a62323", size = 3.5, fontface = "bold") +

 scale_x_continuous(

 limits = c(-2.8, 5.5),

 breaks = c(-2.5, 0, 2.5, 5.0)

 ) +

 theme(axis.title = element_blank()) +

 labs(

 title = "Differentially accessible peaks",

 x = expression("Avg" ~-log[2] ~ "(adj. p-value)"),

 y = expression(-log[10] ~ "(adj. p-value)")

 )+

 theme_classic(base_size = 12) +

 theme(

 plot.title = element_text(hjust = 0.5, face = "bold"),

 axis.line = element_line(color = "black"),

 panel.grid = element_blank()

 )

# Arrange into a panel

panel <- plot_grid(

 p1, p2, p3,

 ncol = 3,

 align = "h",

 axis = "bt",

 labels = c("A", "B", "C"),

 label_size = 12

)

ggsave("plots/figure_panel.tiff", plot = panel, width = 14, height = 5, units = "in")

**H. (Optional) Add PCS as a module score for each cell**

1. If you chose to add the remap binding enrichment scores as an assay in section C, you can proceed to add the PCS as a module score to the remap assay. For this, we would load in the Seurat object with the “remap” assay and subset to epithelial cells first.

# load seurat object with remap assay

obj <- readRDS("data/seurat_obj.rds")

DefaultAssay(obj) <- "ATAC"

# subset to celltype of interest

Tumor_Epi <- subset(obj, subset = celltype == "Epithelial_cells")

Tumor_Epi <- AddModuleScore(

 object = Tumor_Epi,

 features = list(patient_PCS_factors_rna),

 assay = "remap",

 name = 'PCS_factors',

 ctrl = 10,

 seed = 123

)

2. Bin the PCS score into quantiles of low, mid, and high PCS scores.

*Note: While adding a module score, Signac sometimes adds a “1” to the end of the metadata name you specified. If that is the case, like below, make sure you access the right metadata column by adjusting the name.*

Tumor_Epi$persist <- case_when(Tumor_Epi$PCS_factors1 > quantile(Tumor_Epi$PCS_factors1)[4] ~ "High", Tumor_Epi$PCS_factors1 < quantile(Tumor_Epi$PCS_factors1)[2] ~ "Low", TRUE ~ "Mid")

saveRDS(Tumor_Epi, "data/TumorEpi_PCSscore.rds")

**Figure 2.**
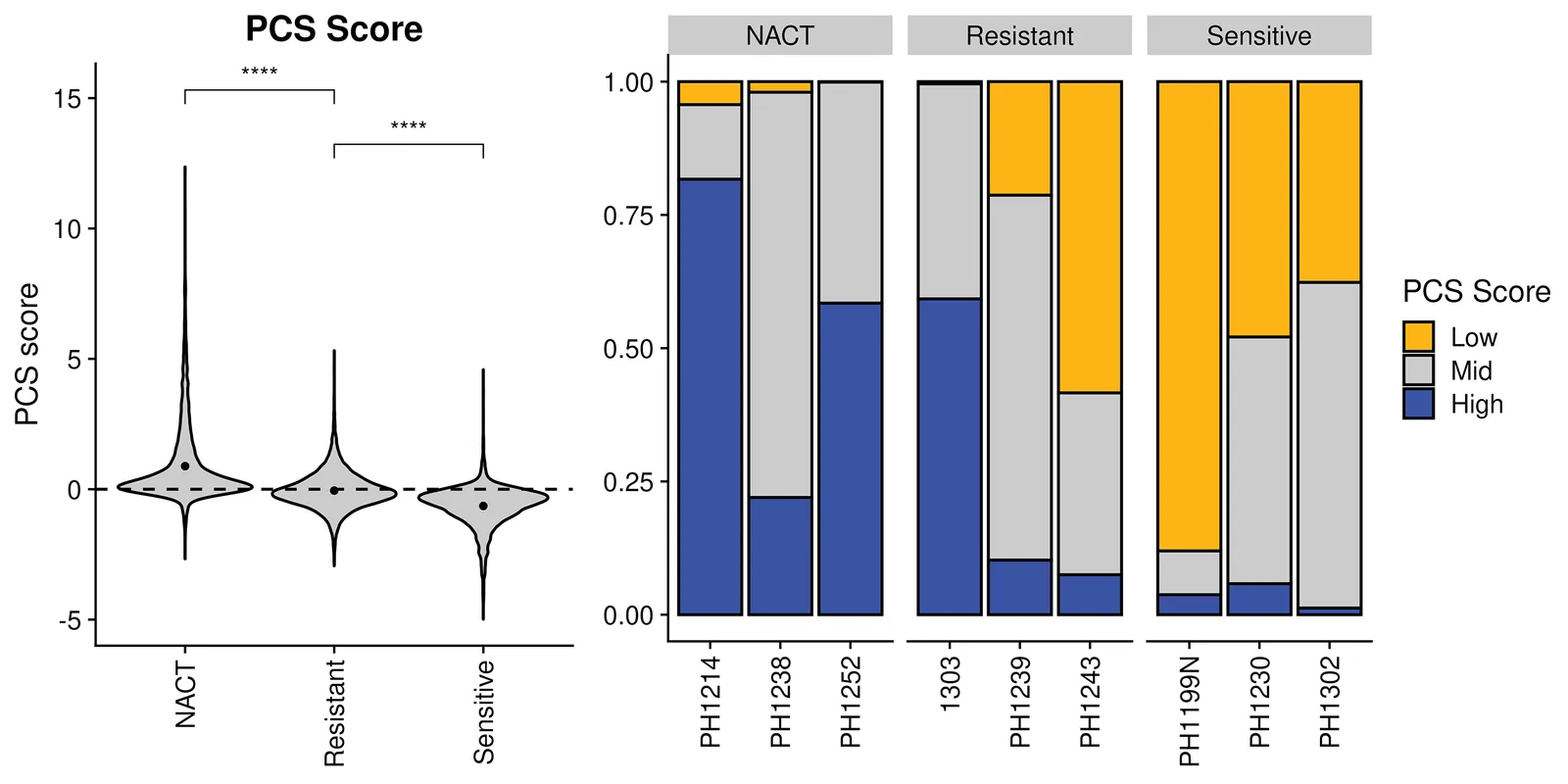
Per-cell persister cell signature (PCS) score. Violin plots (left) showing the distribution of the PCS score per cell between naïve sensitive, naïve resistant, and neoadjuvant chemotherapy (NACT) tumors. The dots represent the mean values for each group. Wilcox test, ****p < 0.0001. Proportion of cells (right) in PCS-high, PCS-mid, and PCS-low states in each patient.

3. Now, we can generate a violin plot of PCS scores in different categories, and a proportion plot to show the proportion of cells in PCS-high, PCS-mid and PCS-low states in each patient (Figure 2).

plotdata <- Tumor_Epi@meta.data %>%

 dplyr::select(persist, orig.ident, grouping2) %>%

 group_by(persist, orig.ident, grouping2) %>%

 mutate(persist = factor(persist, levels = c("Low", "Mid", "High"))) %>%

 summarize(n = n())

table1 <- Tumor_Epi@meta.data %>% dplyr::select(orig.ident, grouping2, PCS_factors1) %>%

 filter(PCS_factors1 != Inf & PCS_factors1 != -Inf)

#violin plot

p1 <- ggplot(table1, aes(x=grouping2, y=PCS_factors1)) +

 geom_violin(colour = "black", fill = "grey80") +

 stat_compare_means(aes(label = ..p.signif..), comparisons = list(c("Sensitive", "Resistant"), c("Resistant", "NACT"))) +

 geom_hline(yintercept = 0, linetype = "dashed", color = "black") +

 stat_summary(fun=mean, geom="point", size=1, color="black") +

 labs(y = "PCS score", title = "PCS Score") +

 theme_cowplot() +

 theme(legend.position = "none", axis.title.x = element_blank(),

 axis.text.x = element_text(angle = 90, vjust = 0.5, hjust=1),

 plot.title = element_text(hjust = 0.5))

#proportion plot

p2 <- ggplot(plotdata, aes(fill=persist, y=n, x=orig.ident)) +

 geom_bar(position="fill", stat="identity", color = "black") +

 facet_grid(cols = vars(grouping2), scales = "free", space = "free") +

 scale_fill_manual(values = c(Low = "#fdb515", Mid = "#cccccb", High = "#3953a4"), name = "PCS Score") +

 theme_cowplot() +

 theme(axis.text.x = element_text(angle = 90, vjust = 0.5, hjust=1), axis.title = element_blank())

combined <- plot_grid(p1, p2, ncol = 2, rel_widths = c(0.6, 1))

ggsave("plots/figure_panel2.tiff", plot = combined, width = 10, height = 5, units = "in")

## Data analysis

The primary output of this protocol is a ranked list of DBFs enriched in the intersection of differentially accessible chromatin regions from two clinical comparisons. Factors ranked at the top have binding sites most significantly overrepresented in the PCS peak set relative to a size-matched background. The intersection of the upregulated peaks in Figure 1A, B is considered as “PCS peaks”, and we define the PCS factors as the top 120 most enriched DBFs in this peak set. Optionally, this list can be refined to the top 100 DBFs by using a threshold for gene expression from scRNA-seq data to remove low-expressed DBFs, as shown in Figure 1C.

## Validation of protocol

This protocol has been used and validated in the following research article:

• Dumbrava et al. [11]. Single-cell resolution of an open chromatin signature in persister tumor cells. *Cell Reports* 45, 116708. https://doi.org/10.1016/j.celrep.2025.116708

Specifically, this protocol was used to generate the results shown in Figure 4I (the PCS dot plot), Figure 4J (PCS score distributions across sensitive, resistant, and NACT groups), and Figure 4K (proportion of PCS-high/mid/low cells per patient). The PCS defined by this workflow independently predicted chemotherapy response in an external metastatic HGSOC cohort (Figure 5C, D) and in patient-derived xenograft models (Figure 7C), and the top PCS factors showed correlated enrichment patterns consistent with cooperative chromatin regulation (Figure 5B).

To validate that this protocol can reproduce the PCS factors shown in the paper, we include the following additional validation step to compare the output factor list against the published PCS factor list from the original paper. In a successful run, the majority of the top 120 unique factors should overlap with the published list, as shown below.

*Note: The original paper uses gene expression data to refine the PCS factor list. For the purpose of this protocol and to make the pipeline more accessible, we have provided the option to follow this pipeline using only snATAC-seq data. Therefore, if not using gene expression data and our customized ReMap file (addons.bed is provided with this protocol), a small amount of non-overlapping factors are expected with the published data. Here, we were able to recover 100 out of the 100 PCS factors from using the top 120 DBFs from the protocol using just chromatin accessibility data, and further refined the list using gene expression data to the top 100 DBFs from the paper.*

> pcs_paper_df <- read.csv("data/PCS_paper_results.csv", header = TRUE)

> pcs_paper_factors <- pcs_paper_df %>% pull(PCS)

> length(setdiff(patient_PCS_factors_rna, pcs_paper_factors))

[1] 0

> length(base::intersect(patient_PCS_factors_rna, pcs_paper_factors))

[1] 100

## General notes and troubleshooting

**General notes**

1. The PCS factor list is subject to ongoing refinement as additional data become available. This protocol provides a generalizable framework for identifying enriched DBFs across clinically defined comparisons and is not limited to the HGSOC context presented here.

2. This protocol was developed and tested on a MacBook Pro (Apple M2 Max, 12-core CPU, 96 GB RAM) running R 4.5.2 and macOS Sequoia 15.7.7, using a representative dataset of 36,213 cells and 318,893 peaks. Object creation and LSI dimensionality reduction (RunTFIDF/RunSVD) completed in ~2 min (peak memory ~4.9 GB). The ReMap chromatin module scoring step (AddChromatinModule), which computes per-cell binding scores against the full ReMap2022 catalog, required ~46 min and ~8.9 GB peak memory. Differential accessibility testing (FindMarkers, both comparisons) was the most memory-intensive step, peaking at ~18.3 GB and completing in ~8 min. The ReMapEnrich enrichment analysis required ~11 min and ~14.7 GB peak memory. The external bedtools intersect step completed in ~1.2 min outside the R session. We recommend a minimum of 16 GB RAM, with 32+ GB recommended for datasets of comparable or larger scale, as memory usage scales primarily with the number of cells, peaks, and reference transcription factors included in the ReMap analysis.

**Troubleshooting**

**Problem 1:** An error occurs when using the subset() function in Seurat:

Error: unable to find an inherited method for function ‘seqinfo’ for signature ‘x = "ChromatinAssay"’

Possible cause: Version incompatibility between Signac and GenomeInfoDb.

Solution: Explicitly define seqinfo before subsetting to a cell type:

setMethod("seqinfo", signature(x = "ChromatinAssay"), function(x) {

 GenomeInfoDb::seqinfo(x@ranges)

})

Tumor_Epi <- subset(obj, subset = celltype == "Epithelial_cells")
